# Recurring genetic variant in LEADS

**DOI:** 10.1002/alz.092647

**Published:** 2025-01-03

**Authors:** Malia C. Rumbaugh, Kelly N. Nudelman, Jeffrey L. Dage, Ani Eloyan, Dustin B. Hammers, Maria C. Carrillo, Bradford C. Dickerson, Gil D. Rabinovici, Liana G. Apostolova, Tatiana M. Foroud, the DIAN/DIAN‐TU Clinical Genetics Committee, and the LEADS Consortium

**Affiliations:** ^1^ Department of Medical and Molecular Genetics, Indiana University School of Medicine, Indianapolis, IN USA; ^2^ Indiana Alzheimer’s Disease Research Center, Indianapolis, IN USA; ^3^ Department of Neurology, Indiana University School of Medicine, Indianapolis, IN USA; ^4^ Department of Biostatistics, Brown University, Providence, RI USA; ^5^ Indiana University School of Medicine, Indianapolis, IN USA; ^6^ Alzheimer’s Association, Chicago, IL USA; ^7^ Massachusetts General Hospital and Harvard Medical School, Boston, MA USA; ^8^ Department of Neurology, Harvard Medical School, Boston, MA USA; ^9^ UCSF Alzheimer’s Disease Research Center, San Francisco, CA USA; ^10^ Department of Neurology, Memory and Aging Center, University of California San Francisco, San Francisco, CA USA; ^11^ Department of Radiology and Imaging Sciences, Indiana University School of Medicine, Indianapolis, IN USA

## Abstract

**Background:**

The Longitudinal Early‐onset Alzheimer’s Disease Study (LEADS) is analyzing the genetic etiology of early onset (40‐64 years) cognitive impairment, including amyloid‐positive early‐onset Alzheimer’s disease (EOAD) and amyloid‐negative early‐onset Alzheimer’s disease (EOnonAD). One goal of this investigation is to identify novel or under‐characterized genetic variants.

**Methods:**

Cognitively impaired (CI) LEADS participants, including amyloid‐positive and amyloid‐negative early‐onset cases, were whole exome or genome sequenced (N = 361). Genetic and copy number variants in *APP*, *PSEN1*, *PSEN2, GRN*, *MAPT* and *C9ORF72* were assessed. Variants in these genes were annotated with Annovar and assessed for potential pathogenicity with criteria including 1,000 genomes population frequency, computational functional prediction, and reported disease occurrence in databases including ClinVar and the Human Gene Mutation Database. Variants were reviewed manually as well as with VarSome and evaluated for pathogenicity using the American College of Medical Genetics criteria. Patient information for carriers of likely pathogenic variants not reported as pathogenic in ClinVar or included in the Dominantly Inherited Alzheimer Network Trial Unit (DIAN‐TU) Trial Eligible list were reviewed.

**Results:**

Three CI participants were found to carry the p.I227L variant in *PSEN1* (3/361, 1%), with no other pathogenic variants identified in these individuals in the six genes analyzed. This variant was previously described in a single individual. It is classified as likely pathogenic by Varsome based on In‐Silico predictors; however, it is listed as “Pathogenicity Not Classified” in AlzForum and as a Variant of Unknown Significance in ClinVar due to the lack of reported cases. The variant is not included in the DIAN‐TU Trial Eligible List. We describe the family history, demographic and clinical data for these three participants.

**Conclusions:**

The *PSEN1* variant p.I227L was identified in three LEADS CI participants and warrants further investigation as a potentially pathogenic variant.

